# sFlt-1/PlGF ratio for prediction of preeclampsia in clinical routine: A pragmatic real-world analysis of healthcare resource utilisation

**DOI:** 10.1371/journal.pone.0263443

**Published:** 2022-02-24

**Authors:** Anne Dathan-Stumpf, Anna Rieger, Stefan Verlohren, Cyrill Wolf, Holger Stepan

**Affiliations:** 1 Department of Obstetrics, University Hospital Leipzig, Leipzig, Germany; 2 Biostatistics, Data Science and Digital Solutions, Roche Diagnostics GmbH, Penzberg, Germany; 3 Department of Obstetrics, Charité–Universitätsmedizin Berlin, Berlin, Germany; 4 Market Access and Health Policy, Roche Diagnostics International Ltd, Rotkreuz, Switzerland; Public Library of Science, UNITED KINGDOM

## Abstract

**Background:**

We investigated the impact of the soluble fms-like tyrosine kinase 1 (sFlt-1)/placental growth factor (PlGF) ratio to predict short-term risk of preeclampsia on clinical utility and healthcare resource utilisation using real-world data (RWD), and compared findings with health economic modelling from previous studies.

**Methods and findings:**

This retrospective analysis compared data from the German population of a multicentre clinical study (PROGNOSIS, n = 203; sFlt-1/PlGF ratio blinded and unavailable for decision-making) with RWD from University Hospital Leipzig, Germany (n = 281; sFlt-1/PlGF ratio used to guide clinical decision-making). A subgroup of the RWD cohort with the same inclusion criteria as the PROGNOSIS trial (RWD prediction only, n = 99) was also included. sFlt-1/PlGF ratio was measured using fully automated Elecsys® sFlt-1 and PlGF immunoassays (cobas e analyser; Roche Diagnostics).

A similar proportion of women in the RWD and PROGNOSIS cohorts experienced preeclampsia (14.95% vs. 13.79%; p = 0.7938); a smaller proportion of women in the RWD prediction only cohort experienced preeclampsia versus PROGNOSIS (6.06%; p = 0.0526). In women with preeclampsia, median gestational age at delivery (weeks) was comparable in the RWD and PROGNOSIS cohorts (34.0 vs. 34.3, p = 0.5895), but significantly reduced in the RWD prediction only cohort versus PROGNOSIS (27.1, p = 0.0038). sFlt-1/PlGF ratio at baseline visit was not statistically significantly different for the RWD and PROGNOSIS cohorts, irrespective of preeclampsia outcome. Hospitalisations for confirmed preeclampsia were significantly shorter in the RWD cohort versus PROGNOSIS (median 1 vs. 4 days, p = 0.0093); there was no significant difference between RWD prediction only and PROGNOSIS (3 days, p = 0.9638). All-cause hospitalisations were significantly shorter in the RWD (median 1 day; p<0.0001) and RWD prediction only (1 day; p<0.0001) cohorts versus PROGNOSIS (3 days).

**Conclusions:**

This study supports the findings of previous studies, showing that routine clinical use of the sFlt-1/PlGF ratio may result in shorter duration of hospitalisations, with potential economic benefits.

## Introduction

Preeclampsia (PE) is a complication of pregnancy that affects approximately 5% of women worldwide [1] and is broadly defined as new or pre-existing hypertension (systolic >140 mmHg and diastolic >90 mmHg) during pregnancy with at least one other organ manifestation that cannot be assigned to any other disease [2–5]. PE is associated with substantial maternal and foetal complications [6–8] and the only cure is delivery; however, the early detection of the disease, alongside appropriate treatment or monitoring, is beneficial for both patients and healthcare professionals [9]. Therefore, a need exists for highly sensitive and specific diagnostic tests to predict or rule out PE [10].

Women with established PE have increased circulating levels of soluble fms-like tyrosine kinase 1 (sFlt-1), an antiangiogenic protein largely produced in the placenta, which is associated with inhibition of vascular endothelial growth factor and placental growth factor (PlGF) signalling in the vasculature [11–16]; hence, development of assays for biochemical markers such as angiogenic factors could be incorporated into more precise definitions of preeclampsia and help to improve prediction and diagnosis of PE [17]. The use of the ratio of sFlt-1 to PlGF has been explored as a prognostic tool to predict the short-term risk of developing PE [18,19] and widely available, automated tests allow rapid and easy measurement of these markers [20].

PROGNOSIS (PRediction of short-term Outcome in preGNant wOmen with Suspected preeclampsIa Study) was an international, prospective, observational study that assessed the use of the sFlt-1/PlGF ratio to predict short-term risk of PE [18]. In two cohorts of 500 (prediction model) and 550 (validation) women, the validation cohort demonstrated that a sFlt-1/PlGF ratio of ≤38 is 99.3% predictive of no PE in the subsequent week, with 80% sensitivity and 78.3% specificity [19]. This was confirmed in a population of 700 Asian women, where a ratio of ≤38 was 98.6% predictive of no PE in the subsequent week [21]. Further analyses of the PROGNOSIS study demonstrated that a ratio of ≤38 could rule out PE for up to 4 weeks, with a negative predictive value of 94.3% [22].

A retrospective, real-world study of 1,117 patients found that women with an adverse pregnancy outcome had a higher median sFlt-1/PlGF ratio (177 vs. 14) compared with women with no adverse outcome, and concluded that use of the ratio, combined with other available information, increased detection of adverse outcomes in women with suspected PE [23]. A further retrospective, real-world study confirmed these results, observing a positive correlation between the sFlt 1/PlGF ratio and severity of placental dysfunction, and a negative association with time to delivery [24]. A recent prospective study also showed that longitudinal changes in sFlt-1 levels improve the prediction of maternal and foetal complications, and interval to delivery in early-onset severe PE [25].

In addition to its clinical importance, use of the ratio could have a measurable, positive economic impact, with cost savings generated through an improvement in prognostic accuracy and a reduction in hospitalisations [26,27]. Within the German cohort of the PROGNOSIS study, it was demonstrated that using a ratio of ≤38 to rule out PE within 1 week could reduce hospital admissions of women with suspected PE from 44.6% to 24.0%, with an expected cost saving of €361 per patient.

Results from clinical studies utilising the sFlt-1/PlGF ratio have now been adopted by learned societies, including the National Institute for Clinical Excellence in the UK [28] and the German Society for Gynecology and Obstetrics [2] on hypertensive gestation disorders, which recommend use of the test to predict PE in the clinical setting.

In this exploratory analysis, we investigated the impact of the sFlt-1/PlGF ratio to predict short-term risk of PE on clinical utility and healthcare resource utilisation in pregnant women based on real-world data (RWD) and compared the findings with health economic modelling from previous studies.

## Methods

### Study design

This was a retrospective analysis of data derived from the German patient population of a multicentre clinical study (PROGNOSIS) compared with RWD from a Medical Centre (University Hospital Leipzig) in Germany, which had broadly comparable clinical endpoints, in order to investigate differences between clinical trial data and routine clinical practice. In both studies, the sFlt-1/PlGF ratio was measured using the fully automated Elecsys® sFlt-1 and PlGF immunoassays on the cobas e analyser (Roche Diagnostics International Ltd). The sFlt-1/PlGF ratio was applied as part of routine practice in the RWD study, but was blinded and therefore not used to guide clinical decision-making in PROGNOSIS. In addition to the PROGNOSIS and RWD cohorts, a subgroup of the RWD cohort with the same inclusion criteria as the PROGNOSIS trial, referred to as ‘RWD prediction only’, was also included in the analyses in order to be more specific and comparable to the PROGNOSIS cohort. A limitation of this approach was that the inclusion criteria in the RWD prediction only group were set retrospectively.

The primary endpoint for this analysis was occurrence of PE within each cohort. Secondary endpoints included maternal age at delivery, gestational age at delivery, sFlt-1/PlGF ratio at baseline, admission to neonatal intensive care unit (NICU), type of birth, and duration of hospitalisations before birth.

### PROGNOSIS study

PROGNOSIS was a prospective, double-blind, non-interventional, observational study in 1,050 women with suspected PE in which serum sFlt-1/PlGF ratios were measured between 24+0 and 36+6 weeks of pregnancy (wop) in order to derive and validate a ratio of serum sFlt-1/PlGF predictive of PE; the methodology and results have previously been described [18,19]. In brief, pregnant women aged ≥18 years with suspected PE were included if they had a gestational age between 24+0 wop and 36+6 wop at the time of first visit (baseline). Women were excluded if they had manifest PE or a confirmed diagnosis of HELLP syndrome. Serum sFlt-1 and PlGF levels were measured weekly using the fully automated Elecsys® sFlt-1 and PlGF immunoassays on the cobas e analyser on Visits 1–5 (Weeks 0–4, respectively) to determine the sFlt-1/PlGF ratio. A sFlt-1/PlGF ratio cut-off of 38 was used to predict the absence of PE within 1 week. PE was defined as a new onset of elevated blood pressure (systolic >140 mmHg and diastolic >90 mmHg) or protein in urine (≥300 mg/24 h), aggravation of previous hypertension or proteinuria, or ≥1 other reason for clinical suspicion of PE taken from a defined list of PE-related symptoms or findings ([29]; **S1 Table**). While PROGNOSIS was a multicentre international study, in this analysis, only data collected at study sites in Germany were included (n = 203; Berlin and Hannover).

### RWD

RWD were collected from a population of women with singleton pregnancies from the University Hospital Leipzig, Germany, where the sFlt/PlGF ratio has been applied in routine clinical practice as a predictive tool for PE since 2009; this population has been previously described by Dathan-Stumpf et al. [24]. The sFlt/PlGF ratio was determined as part of routine clinical practice, with all women with ≥1 determination of the sFlt/PlGF ratio recorded between January and December 2017 included in the cohort. In contrast to PROGNOSIS, women with manifest PE were also included in the RWD cohort; however, only women with the same inclusion criteria as the PROGNOSIS trial were included in the RWD prediction only subgroup. The sFlt-1/PlGF ratio at admission was calculated and additional measurements of sFlt-1/PlGF were recorded for some women after the first determination at admission and before delivery. The sFlt 1/PlGF ratio at admission was defined as normal (<38), intermediate (38–85 for <34 wop; 38–110 for ≥34 wop), or pathologic (>85 for <34 wop; >110 for ≥34 wop) [20]. Ratio cut-offs of >85 (<34.0 wop) and >110 (≥34.0 wop) were used to confirm the diagnosis of a PE. PE was defined, according to the recent International Society for the Study of Hypertension in Pregnancy (ISSHP) definition of 2018 [3], as recent or pre-existing hypertension (>140/90 mmHg) in pregnancy with ≥1 other organ manifestation that could not be attributed to other causes. Data on PE events and secondary endpoints were extracted retrospectively from birth registers, electronic medical records, and ultrasound findings.

### Ethical approval

For PROGNOSIS, the study site provided Ethics Committee/Institutional Review Board approval (10/0503—ZS EK 11 for German sites [30]) of the study protocol and associated documents (participant informed consent, participant information) before the start of the clinical part of the study. All women provided written informed consent before enrolment.

For the RWD cohort, written informed consent for the scientific use of anonymised data was obtained as an institutional standard procedure for each patient. The study was submitted to, and approved by, the Institutional Ethical Committee of the University of Leipzig (IRB00001750; registration number 180/18-ek). All procedures were in accordance with the ethical standards of the responsible committee on human experimentation (institutional and national) and with the Helsinki Declaration of 1975 (in its most recently amended version).

### Statistical analysis

Descriptive statistics for all endpoints are given for the RWD cohort and RWD prediction only subgroup and compared with the PROGNOSIS cohort. As this analysis was a retrospective comparison of RWD with a clinical study subgroup population, RWD were collected between January and December 2017 and there was no prior sample size calculation. Standard statistical methods were used, with the significance level 5% (p = 0.05) for all tests: Fisher’s exact test was used for occurrence of PE, admission to NICU, and type of birth; the Wilcoxon-Mann-Whitney test was used for maternal and gestational age at delivery, sFlt-1/PlGF ratio at baseline, and duration of hospitalisations. Due to the skewed distribution of sFlt-1/PlGF ratios, median (interquartile range [IQR]) values are listed (mean [± standard deviation] are also included in tables for reference); p-values refer to the differences between median values.

## Results

A total of 203 women from study sites in Germany were included from the PROGNOSIS cohort; 281 women were included in the RWD cohort, and of these, 99 had the same inclusion criteria as the PROGNOSIS cohort (**S1 Table**) and were included in the RWD prediction only subgroup analysis.

### Clinically relevant demographics

A similar proportion of women in the RWD and PROGNOSIS cohorts experienced PE (14.95% vs. 13.79%, respectively; p = 0.7938) (**Table 1**); a smaller proportion of women in the RWD prediction only cohort experienced PE compared with those in the PROGNOSIS cohort (6.06% vs. 13.79%), but this difference was not statistically significant (p = 0.0526) (**Table 1**).

**Table 1:** PE events in women with and without PE from the RWD, RWD prediction only and PROGNOSIS cohorts.

Cohort	N	n	Percentage [95% CI]	p-value
**RWD**	281	42	14.95 [10.99–19.66]	0.7938
**RWD prediction only**	99	6	6.06 [2.26–12.73]	0.0526
**PROGNOSIS**	203	28	13.79 [9.37–19.31]	reference

CI, confidence interval; N, total number of women in cohort; n, total number of women with PE event; PE, preeclampsia; RWD, real-world data.

Data were comparable across cohorts for maternal age at delivery, irrespective of the occurrence of PE (**Table 2**). In women without PE, gestational age at delivery was significantly greater in the RWD (p = 0.0021) and RWD prediction only (p<0.0001) cohorts compared with the PROGNOSIS cohort (**Table 2**; **S1 Fig**). In women with PE, median gestational age at delivery was comparable between women in the RWD and PROGNOSIS cohorts (34.0 wop [IQR 8.0] vs. 34.3 wop [IQR 5.0], p = 0.5895), but significantly reduced in women in the RWD prediction only cohort compared with the PROGNOSIS cohort (27.1 wop [IQR 4.0] vs. 34.3 wop [IQR 5.0], p = 0.0038).

**Table 2:** Maternal and gestational age at delivery as well as sFlt-1/PlGF ratio at baseline visit in women with and without PE from the RWD, RWD prediction only and PROGNOSIS cohorts.

Parameter	Cohort	Group	N	Mean	SD	Median	IQR	Min.	Max.	p-value
**Maternal age at delivery (years)**	**RWD**	All	281	30.4	5.6	30.0	7.0	18.0	45.0	0.6908
		No PE	239	30.3	5.7	30.0	7.0	18.0	45.0	0.7634
		PE	42	30.6	5.4	30.0	6.0	18.0	44.0	0.7869
	**RWD prediction only**	All	99	30.8	5.2	30.0	6.0	18.0	44.0	0.3902
		No PE	93	30.6	5.1	29.0	6.0	18.0	44.0	0.6077
		PE	6	34.2	5.2	32.0	8.0	27.0	40.0	0.1529
	**PROGNOSIS**	All	203	30.1	5.6	30.0	8.0	18.0	43.0	reference
		No PE	175	30.1	5.7	30.0	8.0	18.0	43.0	reference
		PE	28	30.3	5.4	30.0	8.0	20.0	42.0	reference
**Gestational age at delivery (weeks)**	**RWD**	All	281	36.8	4.2	38.1	3.0	23.0	41.1	0.0052
		No PE	239	37.5	3.7	39.0	3.0	23.0	41.1	0.0021
		PE	42	32.9	5.0	34.0	8.0	23.0	40.1	0.5895
	**RWD prediction only**	All	99	37.9	3.3	39.0	2.0	25.1	41.1	<0.0001
		No PE	93	38.6	2.2	39.0	2.0	28.1	41.1	<0.0001
		PE	6	28.2	3.4	27.1	4.0	25.1	34.1	0.0038
	**PROGNOSIS**	All	203	36.3	3.8	37.4	4.0	24.3	41.9	reference
		No PE	175	36.8	3.7	38.0	3.0	24.3	41.9	reference
		PE	28	33.6	3.3	34.3	5.0	26.3	39.1	reference
**sFlt-1/PlGF ratio at baseline visit**	**RWD**	All	281	95.8	220.9	22.4	76.2	0.5	2430.7	0.0996
		No PE	239	60.9	181.9	17.6	47.1	0.5	2430.7	0.2246
		PE	42	294.4	306.1	151.9	402.4	4.5	1065.3	0.0582
	**RWD prediction only**	All	99	47.2	137.7	8.0	19.4	0.5	974.5	0.0173
		No PE	93	23.8	60.0	6.3	18.5	0.5	509.7	0.0469
		PE	6	409.2	371.6	317.7	428.0	18.7	974.5	0.1614
	**PROGNOSIS**	All	203	76.4	199.9	13.2	69.7	0.6	1831.1	reference
		No PE	175	60.5	172.7	10.2	45.7	0.6	1831.1	reference
		PE	28	176.2	307.8	117.2	120.5	1.5	1615.4	reference

IQR, interquartile range; Max., maximum; Min., minimum; N, total number of women in cohort; PE, preeclampsia; PlGF, placental growth factor; RWD, real-world data; SD, standard deviation; sFlt-1, soluble fms-like tyrosine kinase 1.

The sFlt-1/PlGF ratio at baseline visit was not statistically significantly different for women in the RWD and PROGNOSIS cohorts, irrespective of PE outcome. The median ratio was significantly lower in all women (with or without PE) (8.0 [IQR 19.4] vs. 13.2 [IQR 69.7], p = 0.0173) or women without PE (6.3 [IQR 18.5] vs. 10.2 [IQR 45.7], p = 0.0469) in the RWD prediction only cohort compared with the PROGNOSIS cohort.

### Duration of PE-related hospitalisation was significantly reduced in the RWD versus the PROGNOSIS cohort

The duration of hospitalisations is shown in **Table 3**; PE-related hospitalisations included patients who were hospitalised due to PE-related symptoms up to delivery, whereas all cause-hospitalisations included patients hospitalised for other reasons (e.g. gastrointestinal infection, shortening of the length of the cervix, premature contractions) in addition to patients who developed late-onset PE.

**Table 3:** Duration of hospitalisations in women with and without PE from the RWD, RWD prediction only and PROGNOSIS cohorts.

Parameter	Cohort	Group	N	Mean duration	SD	Median duration	IQR	Min.	Max.	p-value
**PE-related hospitalisations**	**RWD**	PE	42	3.5	4.5	1	4	0	21	0.0093
	**RWD prediction only**	PE	6	5.0	3.8	3	2	1	12	0.9638
	**PROGNOSIS**	PE	28	7.2	8.5	4	8	0	35	reference
**All-cause hospitalisations**	**RWD**	All	281	2.5	4.5	1	3	0	40	<0.0001
		No PE	239	2.1	4.1	1	2	0	40	<0.0001
		PE	42	4.7	6.0	3	5	0	25	0.0059
	**RWD prediction only**	All	99	2.0	4.8	1	2	0	40	<0.0001
		No PE	93	1.8	4.9	1	1	0	40	<0.0001
		PE	6	5.0	3.8	3	2	1	12	0.4540
	**PROGNOSIS**	All	202	5.5	7.7	3	6	0	62	reference
		No PE	174	5.0	7.5	3	5	0	62	reference
		PE	28	8.4	8.5	6	8	1	35	reference

IQR, interquartile range; Max., maximum; Min., minimum; N, total number of women in cohort; PE, preeclampsia; RWD, real-world data; SD, standard deviation.

In PE-related hospitalisations, women in the RWD cohort had significantly shorter hospitalisations than those in the PROGNOSIS cohort (median 1 day [IQR 4] vs. 4 days [IQR 8], p = 0.0093). There was no significant difference in the median duration of PE-related hospitalisations in the RWD prediction only and PROGNOSIS cohorts (3 days [IQR 2] vs. 4 days [IQR 8], p = 0.9638) (**Table 3**, **S2 Fig**).

All women (with and without PE) in the RWD (median 1 day [IQR 3]; p<0.0001) and RWD prediction only (median 1 day [IQR 2]; p<0.0001) cohorts had significantly shorter all-cause hospitalisations than those in the PROGNOSIS cohort (median 3 days [IQR 6]); this was also true for women without PE in the RWD (median 1 day [IQR 2]; p<0.0001) and RWD prediction only (median 1 day [IQR 1]; p<0.0001) cohorts compared with those in the PROGNOSIS cohort (median 3 days [IQR 5]) as well as for women with PE in the RWD cohort (median 3 days [IQR 5]; p = 0.0059) compared with the PROGNOSIS cohort (median 6 days [IQR 8]) (**Table 3**, **S2 Fig**).

### Clinical outcomes across the RWD and PROGNOSIS cohorts

Significantly more women in the RWD cohort, irrespective of PE status, had a vaginal delivery compared with those in the PROGNOSIS cohort (60.14% [95% CI: 54.16–65.91] vs. 36.45% [95% CI: 29.83–43.48]; p<0.0001), where a greater proportion had caesarean sections (63.55% [95% CI: 56.52–70.17]) (**Table 4**). The same was true with respect to vaginal delivery for all women (with and without PE) (63.64% [95% CI: 53.36–73.07] vs. 36.45% [95% CI: 29.83–43.48]; p<0.0001) and women without PE (65.59% [95% CI: 55.02–75.14] vs. 41.14% [95% CI: 33.77–48.82]; p = 0.0002) in the RWD prediction only cohort, compared with those in the PROGNOSIS cohort; there was no significant difference in mode of delivery in women with PE in the RWD prediction only cohort and the PROGNOSIS cohort (p = 0.1347). The number of NICU events was comparable across all cohorts, irrespective of PE status (**Table 4**).

**Table 4:** Mode of delivery and NICU events in women with and without PE from the RWD, RWD prediction only and PROGNOSIS cohorts.

Cohort	Group	N	Caesarean section		Vaginal delivery		Delivery mode	NICU events		
			n	Percentage [95% CI]	n	Percentage [95% CI]	p-value	n	Percentage [95% CI]	p-value
**RWD**	All	281	112	39.86 [34.09–45.84]	169	60.14 [54.16–65.91]	<0.0001	85	30.25 [24.93–35.99]	0.1219
	No PE	239	84	35.15 [29.10–41.56]	155	64.85 [58.44–70.90]	<0.0001	53	22.18 [17.07–27.98]	0.4625
	PE	42	28	66.67 [50.45–80.43]	14	33.33 [19.57–49.55]	0.0182	32	76.19 [60.55–87.95]	0.0693
**RWD prediction only**	All	99	36	36.36 [26.93–46.64]	63	63.64 [53.36–73.07]	<0.0001	16	16.16 [9.53–24.91]	0.1766
	No PE	93	32	34.41 [24.86–44.98]	61	65.59 [55.02–75.14]	0.0002	10	10.75 [5.28–18.89]	0.1148
	PE	6	4	66.67 [22.28–95.67]	2	33.33 [4.33–77.72]	0.1347	6	100 [54.07–100]	0.0617
**PROGNOSIS**	All	203	129	63.55 [56.52–70.17]	74	36.45 [29.83–43.48]	reference	48	23.65 [17.98–30.10]	reference
	No PE	175	103	58.86 [51.18–66.23]	72	41.14 [33.77–48.82]	reference	33	18.86 [13.35–25.45]	reference
	PE	28	26	92.86 [76.50–99.12]	2	7.14 [0.88–23.50]	reference	15	53.57 [33.87–72.49]	reference

CI, confidence interval; NICU, neonatal intensive care unit; N, total number of women; n, number of women with event; PE, preeclampsia; RWD, real-world data.

### The sFlt-1/PlGF ratio can be used to rule out onset of PE within 1 week

The sFlt-1/PlGF ratio can be used to rule out PE when PE has been shown to be highly unlikely within 1 week; if the sFlt-1 ratio indicates that PE is probable, no rule-out will occur. **Table 5** shows the distribution of how the test was used, with the sFlt-1/PlGF ratio used most frequently to establish PE as highly unlikely and therefore rule out PE within 1 week.

**Table 5:** Distribution of 1-week rule-out in women from the RWD, RWD prediction only and PROGNOSIS cohorts.

PE 1-week rule-out	PE status (1 week)	RWD (N = 281)		RWD prediction only (N = 99)		PROGNOSIS (N = 203)		p-value RWD vs. PROGNOSIS	p-value RWD prediction only vs. PROGNOSIS
		n	Percentage [95% CI]	n	Percentage [95% CI]	n	Percentage [95% CI]		
No rule-out / PE predicted	No PE within 1 week	92	32.74 [27.28–38.57]	13	13.13 [7.18–21.41]	65	32.02 [25.66–38.91]	0.9452	0.0007
Rule-out / no PE predicted	No PE within 1 week	165	58.72 [52.72–64.53]	82	82.83 [73.94–89.67]	128	63.05 [56.02–69.7]	0.3850	0.0007
No rule-out / PE predicted	PE within 1 week	23	8.19 [5.26–12.03]	4	4.04 [1.11–10.02]	8	3.94 [1.72–7.62]	0.0903	>0.9999
Rule-out / no PE predicted	PE within 1 week	1	0.36 [0.01–1.97]	0	0.00 [0.00–3.66]	2	0.99 [0.12–3.51]	0.7766	0.8140

In women presenting with clinical suspicion of PE, a sFlt-1/PlGF ratio of ≤38 has been shown to rule out the onset of PE within 1 week. This table shows the distribution of how the test was used in each cohort.

CI, confidence interval; PE, preeclampsia; RWD, real-world data.

## Discussion

In this study, the sFlt-1/PlGF ratio was a useful tool for predicting short-term PE outcomes in women with singleton pregnancies in a real-world setting and use of the sFlt-1/PlGF ratio in routine clinical practice resulted in significantly shorter duration of hospitalisations of women with PE.

The financial burden of PE is difficult to calculate and for decades has been mainly driven by foetal prematurity and the resulting costs. Any financial assessment can only be an estimation restricted to immediate costs. Due to the low predictive value of the measurement of hypertension and proteinuria in women with PE [10,19], assays of biochemical markers such as angiogenic factors [17] have the potential to aid prognosis and diagnosis of the condition. A study by Delahaije et al. found that 80% of total costs in women with PE or HELLP syndrome were maternal admissions and outpatient visits [31]. Since the duration of hospitalisations of pregnant women can easily be measured, it is a good surrogate for immediate financial impact. By significantly reducing duration of hospitalisations of women with PE, use of the sFlt-1/PlGF ratio in routine clinical practice benefits both the women themselves and healthcare providers by reducing healthcare resource utilisation and potentially reducing economic burden.

The PROGNOSIS and RWD cohorts in this study were broadly comparable in terms of clinically relevant demographics and measurable outcomes, including number of PE cases, and maternal and gestational age at delivery. While in the RWD prediction only cohort gestational age at delivery was significantly reduced in women with PE, and the sFlt-1/PlGF ratio was significantly lower in all women, compared with the PROGNOSIS cohort, it should be noted that these differences could be attributable to the relatively small number of women with PE in the RWD prediction only cohort (n = 6). Despite comparable inclusion criteria, it can be assumed that the RWD prediction only cohort had more cases with early manifestation and shorter prolongation than the PROGNOSIS cohort. With later manifestation and higher overall gestational age, more women had the opportunity to develop PE in the PROGNOSIS cohort than the RWD prediction only cohort. A larger number of PE cases in the RWD prediction only cohort with more late-onset manifestation might have led to an approximation of the results of PROGNOSIS cohort. Since number of PE cases and gestational age between PROGNOSIS and RWD cohorts were comparable, it is unsurprising that the number of NICU events was also comparable across cohorts. The similarity in gestational age at delivery and NICU admissions between PROGNOSIS and RWD cohorts likely results from the fact that angiogenic factors in all cases were not used to determine the time point of delivery in all cases. In the RWD cohort, the implementation of the sFlt-1/PlGF ratio did not lead to a prolongation of the pregnancy, which was expected. Therefore, knowledge of the ratio can help stratify patient management (e.g. by employing step-down care) but does not improve disease outcome and natural course of the disease directly. Additionally, while significantly more women in the RWD cohort had a vaginal delivery compared with those in the PROGNOSIS cohort, this was most likely due to local guidelines, rather than PE status.

The results from the RWD cohort correlate with a number of other studies that have evaluated the potential economic benefit of the sFlt-1/PlGF ratio as a predictive measure of PE. Using an Excel-based cost-effectiveness model to evaluate the economic impact of the sFlt-1/PlGF ratio using ratio test characteristics and healthcare resource utilisation derived from the PROGNOSIS study, Vatish et al. demonstrated that use of the sFlt-1/PlGF ratio in clinical practice could result in cost savings generated through an improvement in diagnostic accuracy and reduction in hospitalisation [26]. In addition, Schlembach et al. adapted the cost-effectiveness model to evaluate the economic impact of the sFlt-1/PlGF ratio from the perspective of the German healthcare system. Using data from the German population of the PROGNOSIS study, they demonstrated that use of the ratio of ≤38 rule helps to reduce unnecessary hospitalisation of women with a low risk of PE, which could translate into cost savings [27].

Identifying the risk of PE through use of the sFlt-1/PlGF ratio has particular clinical importance as treatment and management can reduce the risk of adverse maternal and foetal health outcomes [23]. The use of the sFlt-1/PlGF ratio has been demonstrated, particularly through data from the PROGNOSIS study, to be an effective negative predictor for risk of developing PE, for up to 4 weeks [19,22], and has also been demonstrated to have comparable accuracy in a real-world setting to that in a clinical study setting for accurately ruling out PE. Dathan-Stumpf et al. demonstrated in a study of 283 singleton pregnancies with ≥1 determination of the ratio that there is a positive correlation between the sFlt-1/PlGF ratio and severity of placental dysfunction, and a negative association with gestational age at delivery, birth weight and time to delivery [24]. The current study showed that the sFlt-1/PlGF ratio was most frequently used to rule out PE within 1 week, but no analyses regarding the test accuracy or sensitivity were included.

In this study, the RWD cohort reflected the findings of previous clinical trials on the use of the sFlt-1/PlGF ratio in routine clinical practice and the possible associated economic benefit. However, the RWD cohort was retrospective, which could introduce a source of bias, particularly with regard to allocation to the RWD prediction only cohort. Additionally, the RWD prediction only cohort contained only a small number of PE cases (n = 6), which could affect the precision of the analyses related to PE cases. Interventional trials should follow to prospectively show the superiority of including the ratio into the clinical work-up of suspected PE over the standard of care to reduce maternal and foetal morbidity and mortality; cost-effective analyses should also be undertaken to quantify any economic benefit.

## Conclusions

This study supports previous findings on the positive economic implications of the routine clinical use of the sFlt-1/PlGF ratio. In addition, the sFlt-1/PlGF ratio is easy to measure and utilisation of the ratio results in shorter duration of hospitalisations, which in turn has a potential economic benefit.

## Supporting information

S1 Fig Gestational age at delivery in the RWD, RWD prediction only and PROGNOSIS cohorts. The median of each data group is depicted with a thick line; mean values are represented by diamonds. The width of the boxplot corresponds to the number of women in each group. PE, preeclampsia; RWD, real-world data; wop, weeks of pregnancy.
(TIF)

S2 Fig Duration of (A) PE-related and (B) all-cause hospitalisations in the RWD, RWD prediction only and PROGNOSIS cohorts. Note: an additional data point was recorded at 125 days in the PROGNOSIS no PE group but the graph has been clipped to allow more detail to be shown. The median of each data group is depicted with a thick line; mean values are represented by diamonds. The width of the boxplot corresponds to the number of women in each group. PE, preeclampsia; RWD, real-world data.
(TIF)

S1 Table Criteria contributing to suspicion of clinical diagnosis of PE (Hund *et al*. 2014). *The presence of at least one of these clinical criteria for suspicion of PE is required for inclusion in the study.^†^Does not need to be defined hypertension (≥140 mmHg systolic and/or ≥90 mmHg diastolic). ^‡^Does not need to be defined proteinuria–any protein in the urine is sufficient. PE, preeclampsia. Table reproduced under the terms of the Creative Commons Attribution License from Hund M, Allegranza D, Schoedl M, et al. Multicenter prospective clinical study to evaluate the prediction of short-term outcome in pregnant women with suspected preeclampsia (PROGNOSIS): study protocol. BMC Pregnancy Childbirth. 2014; 14:324. https://doi.org/10.1186/1471-2393-14-324.
(DOCX)
