# Supplementary figures and images for: sFlt-1/PlGF ratio for prediction of preeclampsia in clinical routine: A pragmatic real-world analysis of healthcare resource utilisation
Source: PLoS One. 2022 Feb 24;17(2):e0263443. doi: 10.1371/journal.pone.0263443 (PMC8870556; doi:10.1371/journal.pone.0263443)

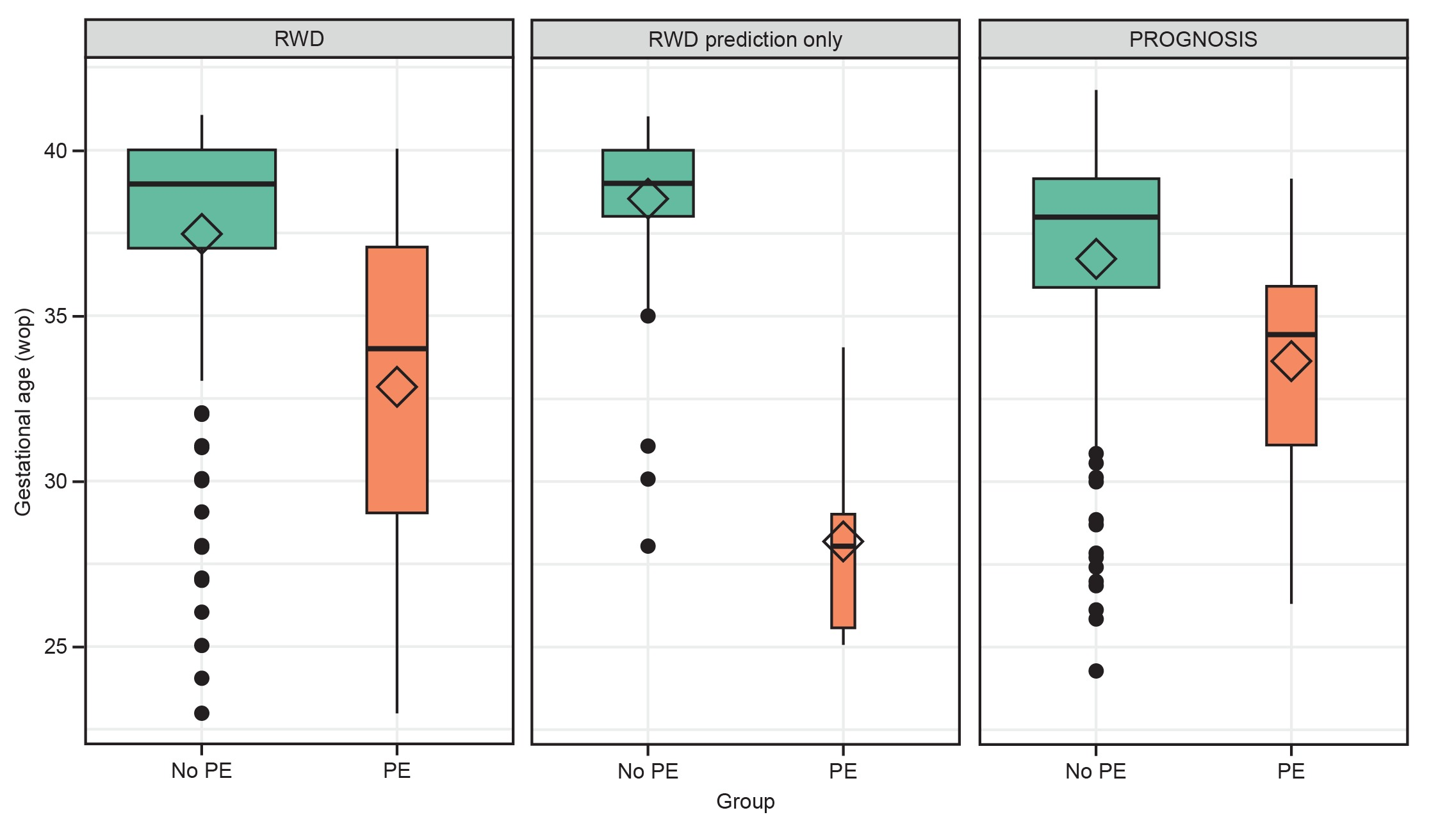

Supplement: S1 Fig — The median of each data group is depicted with a thick line; mean values are represented by diamonds. The width of the boxplot corresponds to the number of women in each group. PE, preeclampsia; RWD, real-world data; wop, weeks of pregnancy. (TIF) [file pone.0263443.s001.tif]

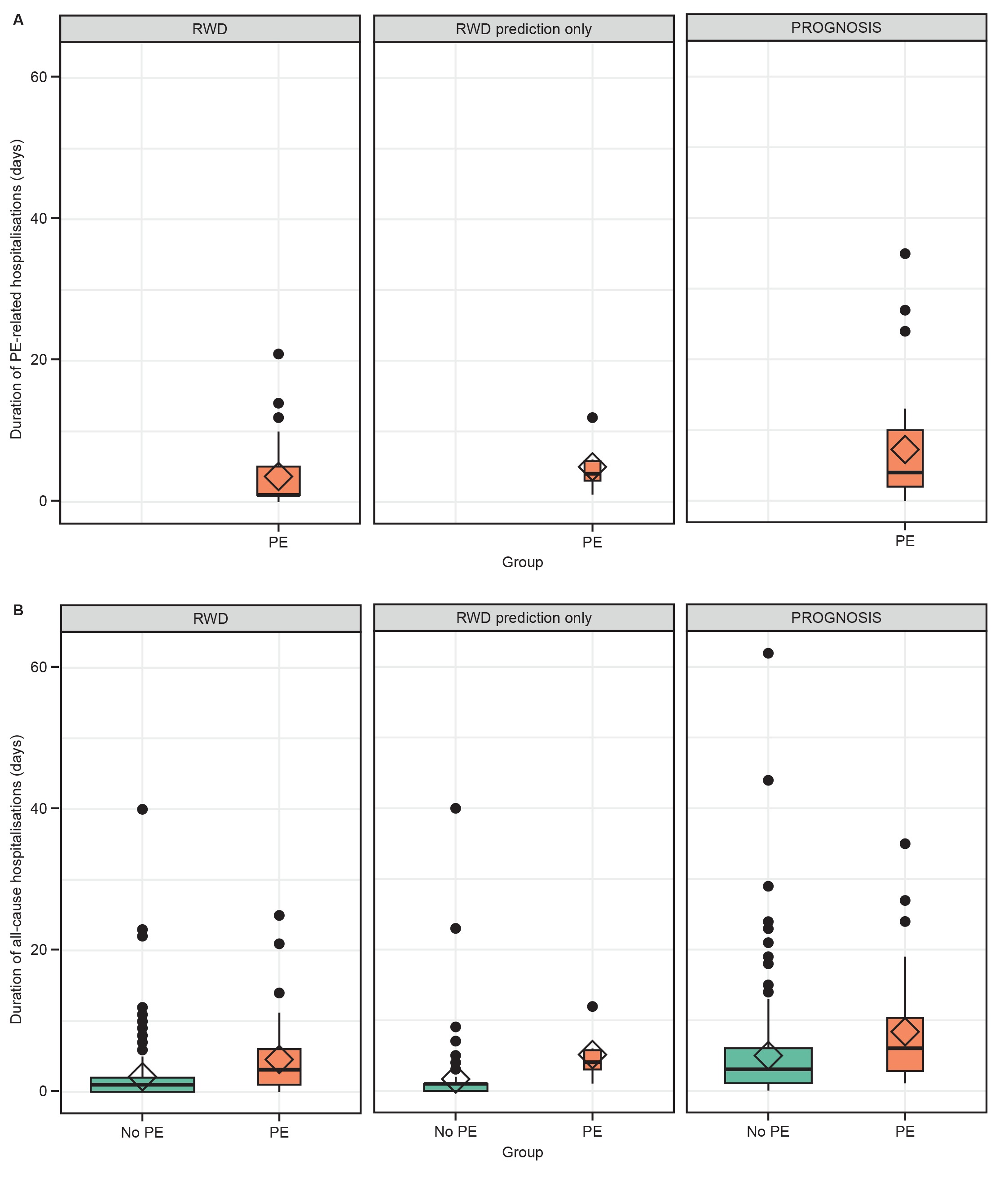

Supplement: S2 Fig — Duration of (A) PE-related and (B) all-cause hospitalisations in the RWD, RWD prediction only and PROGNOSIS cohorts. Note: an additional data point was recorded at 125 days in the PROGNOSIS no PE group but the graph has been clipped to allow more detail to be shown. The median of each data group is depicted with a thick line; mean values are represented by diamonds. The width of the boxplot corresponds to the number of women in each group. PE, preeclampsia; RWD, real-world data. (TIF) [file pone.0263443.s002.tif]
