# Supplementary material for: sFlt-1/PlGF ratio for prediction of preeclampsia in clinical routine: A pragmatic real-world analysis of healthcare resource utilisation
Source: PLoS One. 2022 Feb 24;17(2):e0263443. doi: 10.1371/journal.pone.0263443 (PMC8870556; doi:10.1371/journal.pone.0263443)
Supplement: S1 Table — *The presence of at least one of these clinical criteria for suspicion of PE is required for inclusion in the study.†Does not need to be defined hypertension (≥140 mmHg systolic and/or ≥90 mmHg diastolic). ‡Does not need to be defined proteinuria–any protein in the urine is sufficient. PE, preeclampsia. Table reproduced under the terms of the Creative Commons Attribution License from Hund M, Allegranza D, Schoedl M, et al. Multicenter prospective clinical study to evaluate the prediction of short-term outcome in pregnant women with suspected preeclampsia (PROGNOSIS): study protocol. BMC Pregnancy Childbirth. 2014; 14:324. https://doi.org/10.1186/1471-2393-14-324. (DOCX) [file pone.0263443.s003.docx]

**S1 Table. Criteria contributing to suspicion of clinical diagnosis of PE (Hund *et al.* 2014).**

| **Clinical signs and symptoms*** | |
| --- | --- |
| a. New onset of elevated blood pressure^†^ | |
| b. Aggravation of pre-existing hypertension | |
| c. New onset of protein in urine^‡^ | |
| d. Aggravation of pre-existing proteinuria | |
| e. One or more other reason(s) for clinical suspicion of PE (see i. and ii.) | |
| **i. PE-related symptoms:** | 1. Epigastric pain |
|  | 2. Excessive oedema/severe swelling (face, hands, feet) |
|  | 3. Headache |
|  | 4. Visual disturbances |
|  | 5. Sudden weight gain (>1 kg/week in the third trimester) |
| **ii. PE-related findings:** | 1. Low platelets |
|  | 2. Elevated liver transaminases |
|  | 3. (Suspected) intrauterine growth restriction |
|  | 4. Abnormal uterine perfusion detected by Doppler sonography with mean pulsatility index >95th percentile in the second trimester and/or bilateral uterine artery notching |

*The presence of at least one of these clinical criteria for suspicion of PE is required for inclusion in the study.

^†^Does not need to be defined hypertension (≥140 mmHg systolic and/or ≥90 mmHg diastolic).

^‡^Does not need to be defined proteinuria – any protein in the urine is sufficient.

PE, preeclampsia.

Table reproduced under the terms of the Creative Commons Attribution License from Hund M, Allegranza D, Schoedl M, et al. Multicenter prospective clinical study to evaluate the prediction of short-term outcome in pregnant women with suspected preeclampsia (PROGNOSIS): study protocol. BMC Pregnancy Childbirth. 2014; 14:324. https://doi.org/10.1186/1471-2393-14-324.
